# Comparison of five single-file systems in the preparation of severely curved root canals: an ex vivo study

**DOI:** 10.1186/s12903-022-02668-3

**Published:** 2022-12-28

**Authors:** Yina Liu, Meizhi Chen, Weilong Tang, Chang Liu, Minquan Du

**Affiliations:** 1 The State Key Laboratory Breeding Base of Basic Science of Stomatology (Hubei-MOST) and Key Laboratory of Oral Biomedicine Ministry of Education, School and Hospital of Stomatology, Wuhan University, Wuhan, China; 2 Department of Preventive Dentistry, Hospital of Stomatology, Wuhan University, Hongshan District, Luo-Yu Road 237th, 430075 Wuhan, Hubei China

**Keywords:** Canal preparation, NiTi instrumentation, Single-file system, Apically debris extrusion, Shaping ability, Cleaning ability, Micro-CT, SEM

## Abstract

**Background:**

The ex vivo study is to compare the root canal preparation outcomes achieved by five nickel–titanium single-file instrumentation systems (M3-L, Reciproc Blue, V-Taper 2H, WaveOne Gold, XP-endo Shaper) in severely curved molar root canals.

**Methods:**

A total of 60 root canals were selected from extracted human molar teeth with curvatures ranging from 25° to 50° and divided into five groups based on the instrumentation system employed (n = 12). Before and after root canal preparation, a Micro-CT scan was taken, and pre- and post-operative data were analyzed to evaluate the following parameters: volume increment of root canals (VI), untouched root canal areas (UTA), and canal transportation (CT). Apically extruded debris (AD) was collected during preparation. After that, all samples were separated into two parts and examined respectively by scanning electron microscope (SEM) to assess cleaning ability. Data were statistically analyzed with ANOVA (UTA, AD, VI) or Kruskal–Wallis test (CT, SEM-score), the level of significance was set at α = 0.05.

**Results:**

There were no significant differences between the five systems regarding the AD, VI, and UTA parameters (*P* > 0.05). In terms of CT, no significant difference was noted at the straight section of canal and apical levels, while XP-endo Shaper showed less canal transportation than M3-L at the level of curved vertex (*P* < 0.05), and the centering ability of V-Taper 2H was significantly better than WaveOne Gold at the initial point of bending (*P* < 0.05). Debris and smear layers were present on the canal walls of all specimens, the apical thirds of the canal presented higher SEM scores than the coronal thirds in all groups (*P* < 0.05). Reciproc Blue and XP-endo Shaper showed fewer smear scores than WaveOne Gold in the apical thirds (*P* < 0.01 and *P* < 0.05, respectively), and no statistical difference was found between other groups in the middle and coronal thirds.

**Conclusion:**

The five single-file systems evaluated performed equally in apically debris extrusion, dentin removal, and untouched root canal areas, while XP-endo Shaper and V-Taper 2H resulted in less canal transportation compared to M3-L and WaveOne Gold. Regarding cleaning ability, Reciproc Blue and XP-endo Shaper were associated with less smear layer than WaveOne Gold in the apical thirds.

**Supplementary Information:**

The online version contains supplementary material available at 10.1186/s12903-022-02668-3.

## Background

Thorough cleaning of root canals is the key step in root canal therapy and is considered critical for successful endodontic treatment because of the anatomical complexity of root canal systems [1–4]. In particular, the curved root canal has so far been a challenge to dentists. Studies have shown that most root canals of molars have some degree of curvature at the apical and cervical thirds, to a certain extent, irrespective of the plane of analysis [5]. Curved root canal preparation presents a greater incidence of procedural errors including root canal transportations, root perforations, and separation of instruments than straight canals [6]. Besides, it is pertinent to note that debris removal is much more difficult in the curved regions, thus leaving certain areas of root canal walls untouched, which may harbor bacteria and result in postoperative endodontic disease [7].

Compared with traditional stainless steel manual instruments, the nickel–titanium file system is superior in maintaining the original canal path to the greatest extent [8], which is eminently suitable for the preparation of curved root canals. However, the files of the original NiTi instruments are stiff and remain vulnerable to fracture due to cyclical and torsional failure [9]. Over the last decades, to circumvent such limitations, the nickel–titanium instrument has made great progress [10]. To improve flexibility and cyclic fatigue resistance, the manufacturers have sought innovation in metallurgy, instrument design, and movement strategies such as various thermo-mechanical treatments, different cross-sectional designs, and new manufacturing processes, leading to a great variety of these endodontic instruments. Among them, the single-file system has been widely used because it greatly enhanced the efficiency of clinical work, thus relieving the patient’s pain and the work intensity of dentists [11, 12]. Therefore, it is necessary to evaluate the preparation outcome of these various types of single-file systems in curved canals to provide guidance for the dentists.

WaveOne Gold (WOG, Dentsply Sirona, Switzerland), and Reciproc Blue (RB, VDW, Germany) are two popular nickel–titanium single-file systems using reciprocating motion. Their unique surface color comes from a proprietary thermal treatment process, which gave them more flexibility and resistance to cyclic bending fatigue than conventional NiTi instruments. XP-endo Shaper (XP, FKG Dentaire SA, Switzerland) is a single-file system newly introduced which is manufactured from MaxWire alloy. It’s claimed that the unique patent design of the instrument can accomplish a preparation of at least 30/04 in size from the original size while maintaining the root canal anatomy and avoiding canal transportation [13]. V-Taper 2H (VT, SSWhite, America) and M3-L (ML, Yirui, China) are CM wire-based NiTi rotary systems. This kind of wire has a controlled memory effect and is extremely flexible. The V-Taper 2H system is an updated version of the traditional NiTi instrument V-Taper 2, with the same cross-section and variable taper [14]. The M3-L system has a traditional double S-shaped cross-section with a unique longitudinal cutting plane, which can increase the space for debris removal. In terms of the mechanical property, all the five systems have good elasticity and flexibility, which are suitable for the preparation of severely curved root canals.

Up to now, a certain number of studies have been performed to assess the canal preparation outcomes of WOG and RB from different aspects. However, literature about XP, VT, and ML were scarce, and there is a lack of research that has developed a comprehensive comparison between these five single-file systems concerning both the shaping ability and cleaning efficiency in preparing severely curved root canals. Therefore, the objective of this study was to assess the apical debris extrusion, shaping, and cleaning ability of the five single-file instrumentation systems above in severely curved molar root canals, using micro-CT scan technology and scanning electron microscopy. The null hypothesis tested was no difference existed between the five systems in severely curved root canal preparation.

## Materials and methods

### Specimen selection and preparation

The sample size was calculated by an effect size of 0.6 determined based on a previous study [15], an alpha-type error of 0.05, and a study power of 0.95, using the F tests family and a priori power analysis (G*Power 3.1.9.3; Heinrich Heine University, Dusseldorf, Germany). The estimated sample size was 60 specimens in total.

Human permanent molar teeth extracted for reasons unrelated to this study were collected and preliminary scanned by micro-CT (SkyScan1176; Bruker, Kontich, Belgium). Finally, 60 of the 100 molars were selected for the study with the following criteria (Table 1). The molars were scanned at 80 kV and 309µA using a pixel size of 9 μm. 180° rotation around the vertical axis and a 0.1-mm-thick copper plus aluminum filter were selected. After scanning, the data were reconstructed using NRecon v.1.18.8.0 software (Bruker, Kontich, Belgium), with smoothing of 5, ring artifacts reduction of 12, beam hardening correction of 30% to distinguish the density of dentin, and presenting the true internal structure of root canal. Region of interest (ROI) was established from the furcation level to the apex of the root, resulting in 600–700 cross-sections per specimen. For each tooth, only one qualified root canal was selected, and the maximum curvature of each root canal was recorded. All the molars were cleaned using an ultrasonic cleaner to remove the periodontal membrane and calculus and stored in 1% chloramine-T (Sinopharm Chemical ReagentCo., Ltd, China) solution at 4 ℃ for the next procedure [16].

**Table 1:** The exclusion and inclusion criteria of specimen

Inclusion criteria	Exclusion criteria
a. Fully formed apex without fracture.	a. Long oval-shaped canals ( the ratio of the long to short canal diameter was > 2).
b. One independent severely curved root canal with a curvature of 25° to 50° according to the Schneider method [17].	b. Root resorption (internal, external, or apical) or calcifications.
	c. Previous endodontic treatment.

Crown access was obtained using diamond burs according to the morphology of the pulp chamber, the canal orifices were located and confirmed with a #10 K-file (VDW, Germany), and the model number of the initial apical file (the K-file whose tip diameter is the same as the apical foramen diameter) was determined and registered as the size of the apical foramen. 60 specimens were matched to create 12 groups of 5 teeth based on their angle of curvature and the size of the apical foramen to enable homogeneous specimen distribution in each experimental group. Then, the 5 teeth from each group were randomly allocated to 5 experimental groups (n = 12) according to the different systems they used: ML, RB, VT, WOG, and XP group. The degree of homogeneity (baseline) between groups was statistically confirmed at a significance level of 5% (*P* > 0.05, 1-way analysis of variance test) (Table 2).

**Table 2:** Characteristics of curved root canal teeth per group (n = 12)

System	Curvature (°)			Size of initial apical file		
	Mean ± SD	Min	Max	Mean ± SD	Min	Max
M3-L	35.58 ± 8.57	25	50	9.50 ± 0.90	8	10
Reciproc Blue	35.33 ± 7.66	25	50	9.50 ± 0.90	8	10
V-Taper 2H	34.92 ± 7.88	25	49	9.67 ± 0.78	8	10
WaveOne Gold	35.42 ± 8.07	25	48	9.33 ± 0.98	8	10
XP-endo Shaper	37.75 ± 8.27	27	50	9.33 ± 0.98	8	10
*P* value (ANOVA)	0.92			0.89		

### Root canal preparation

To avoid apical transportation before preparation, the working length (WL) was determined by introducing a pre-curved #06 K-file into the canal until it was visible at the main apical foramen and then withdrawing it 0.5 mm. A glide path was established after manual preparation up to ISO 15/0.02 using stainless K-files, and no coronal expansion was conducted to prevent interfering with coronal transportation. Then, root canal preparations were performed using the different nickel–titanium systems in each system according to the manufacturer’s instructions. In the ML group, the L2 file (25/06.5) was used in rotary motion at a speed of 350 rpm and torque of 2.5 Ncm; in RB group, the R25 file having a tip size of 25 and a 0.08 taper over the first 3 mm was utilized in a reciprocating mode; in VT group, the V25/06 file was used at 350 rpm and 2 Ncm in rotary motion; in WOG group, the primary file (25/07 red) was employed with a reciprocating mode; in XP group, the file was operated at 800 rpm and 1 Ncm in rotary motion.

All of the files were gently inserted into the root canals and applied with light up-and-down movements with light apical pressure at a distance of 1 mm (3 to 5 up-and-down strokes every time). It was necessary that the canals remain moist during preparation. After every 3 to 5 up-and-down strokes, if the WL was not achieved, the file should be withdrawn from the root canal while rotating, followed by irrigation of the canal with 2 mL 2.5% NaOCl (Longly, Wuhan, China) for 30 s. Then the patency was rechecked with a #6 K-file, and after that, the preparation was restarted again. The operations above were repeated until the WL was reached. In the XP group, the file was moved up and down over the entire length five times after it reached the WL to achieve the final size of approximately 30/04. Final irrigation was performed by rinsing 5 mL of 2.5% NaOCl solution for 1 min at a distance of 1 mm from the WL. A total of 15 mL of flushing fluid was used for each root canal using a syringe with a 30-guide-vented NaviTip irrigation needle (Ultradent, South Jordan, America). To eliminate bias owing to instrument fatigue, each fresh file was used to prepare only one canal. All instrumentation operations were conducted by a single endodontist with 10 years of clinical experience in a 37 ℃ incubator (TAISITE, Tianjin, China), and no accidents or errors like instrument fractures occurred during root canal preparation in the 5 groups.

### Debris assessment

**Fig. 1:**
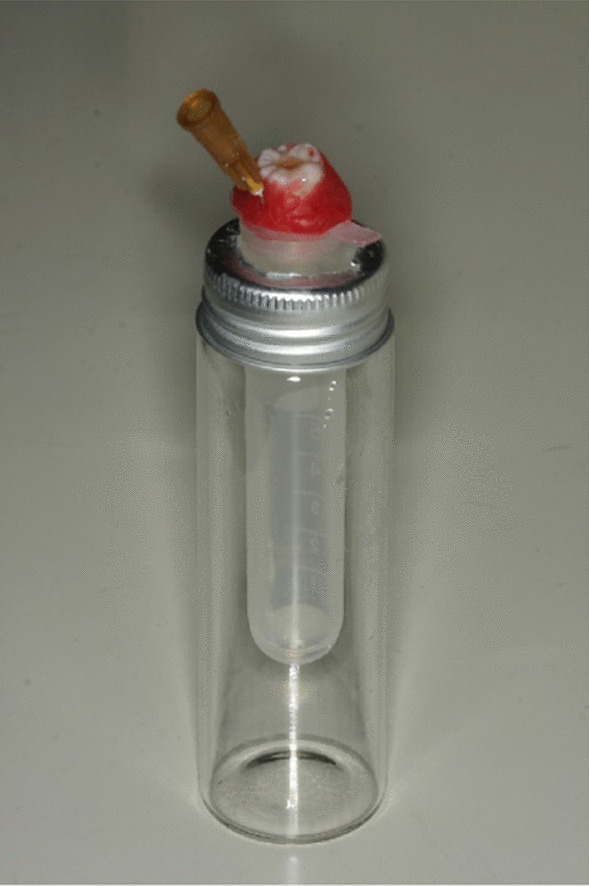
Apically extruded debris collection device

The whole preparation process described above was performed in an apically extruded debris collection device adapted from a past report [18] (Fig. 1). The tooth was fixed with wax at the level of cementoenamel junction (CEJ) in a circular opening made in the separated cap of a 10 mL Eppendorf tube large enough to hold it. Then a 50 mL glass bottle with a rubber stopper was prepared, digging a round hole in the rubber stopper with the same diameter as the Eppendorf tube, and inserting the tube into the glass bottle to avoid fouling during preparation and interference with the weighing result. Afterward, a 5 mL disposable syringe needle was inserted into the tube cap to balance the internal and external pressures. Finally, the glass bottle is wrapped in tinfoil to prevent the operator from seeing the debris extrusion.

Before preparation, the Eppendorf tube was weighed by cap removal (average weight three times, precision 0.0001 g) using the precision balance (CP214C, OHAUS, America) (Additional file 1). After preparation, the debris adhering to the root surface was collected by washing the root with 1ml distilled water while in the tube. Being dried in an oven at 110 °C for 5 h, the Eppendorf tube containing dentin debris was weighed by cap removal again (average weight three times, precision 0.0001 mg). The difference in weighing results was calculated, which was the amount of apically extruded debris (AD).

### Evaluation of shaping ability

**Fig. 2:**
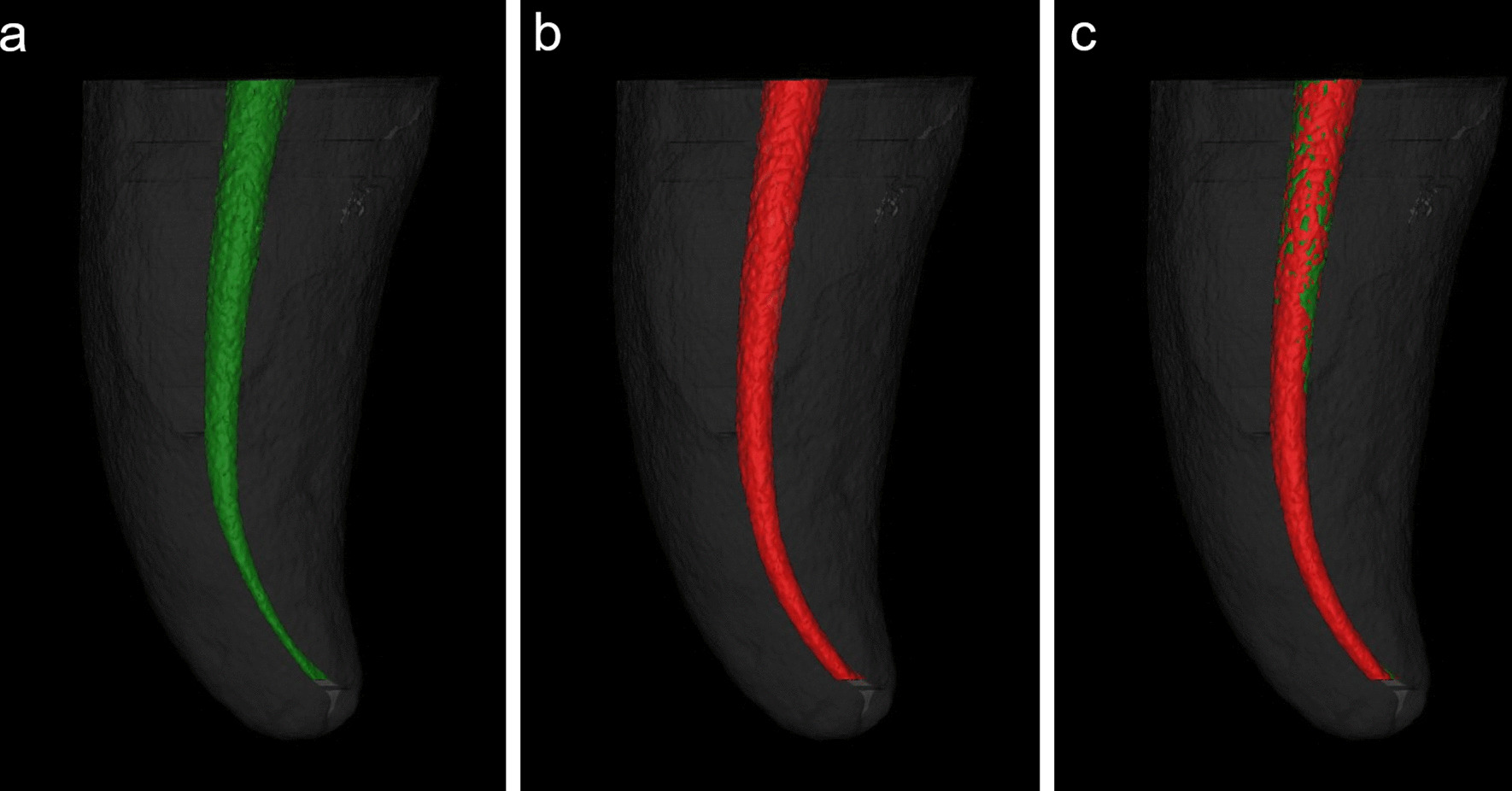
Three-dimensional reconstruction of the root canal. **a** Preoperative root canal (in green). **b** Postoperative root canal (in red). **c** The superimposition of root canals before and after preparation, the green regions on the surface presented the untouched areas

**Fig. 3:**
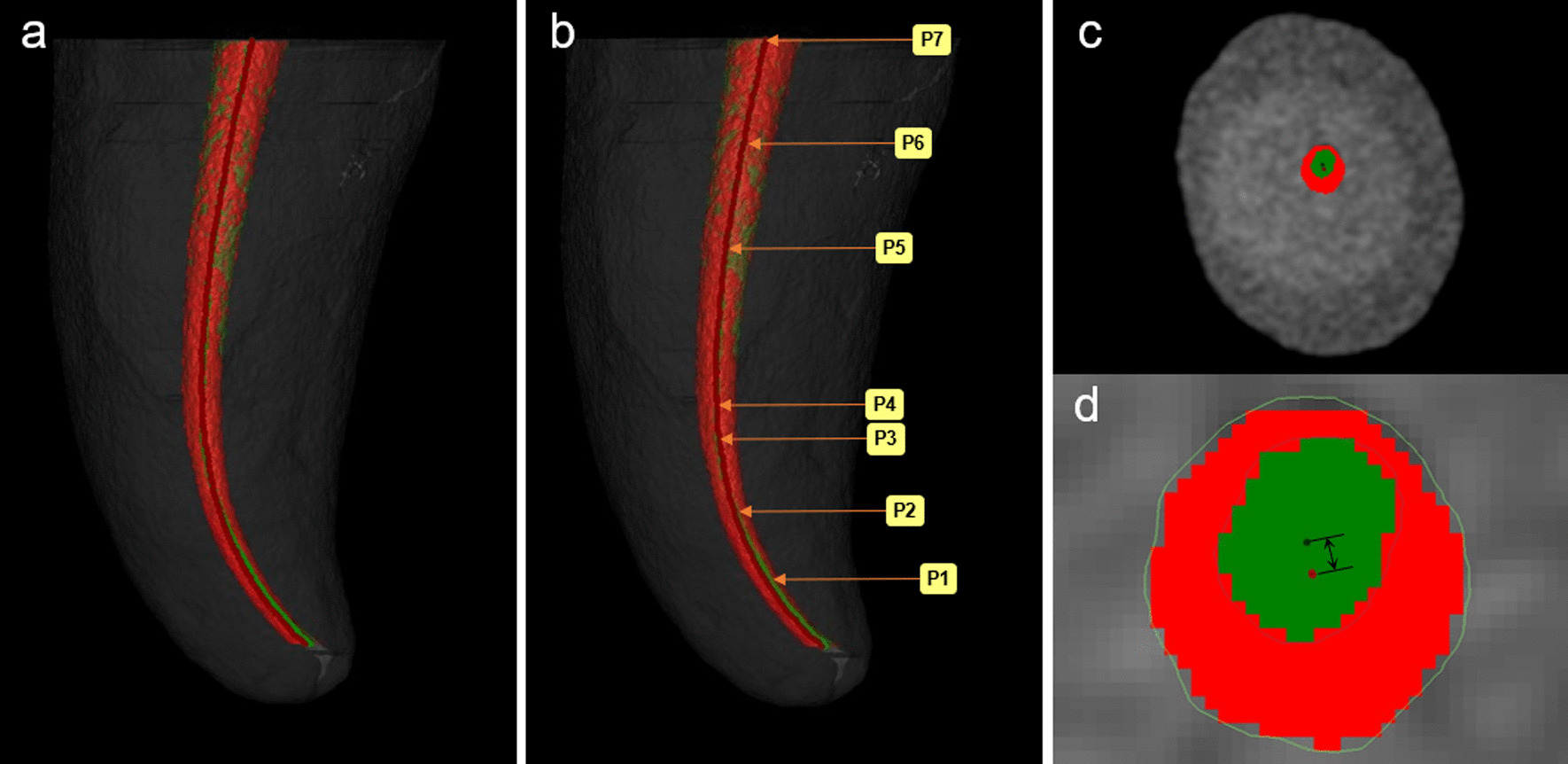
Three-dimensional reconstruction of the root canal. **a** The centerlines of the root canal before (in green) and after preparation (in red). **b** P1-P7 were selected in advance. **c**, **d** The representative photo of measurement of canal transportation in P1

The specimens were rescanned and reconstructed after canal preparation, with the same parameters as the initial scan. The pre- and post-preparation data were imported into Mimics Research v.20.0 software (Materialise, Leuven, Belgium) for 3D reconstruction and analysis. The pre- and post-preparation root canals were visualized and colored green and red, respectively, and the volumes of the pre- and post-preparation canals were measured, allowing the percentage of increment in root canal volume (%VI) to be calculated. The Mimics “Align” function was employed to calibrate and superimpose the root canal 3D models before and after preparation (Fig. 2). The untouched root canal areas (UTA) could be displayed (the green areas) and the percentage of UTA could be estimated using the formula: The number of static voxels × 100/total number of surface voxels. For analysis of root canal transportation, the “Fit Centerline” function of Mimics was used to fit the axial centerlines of root canals before and after instrumentation, canal transportation (CT) could be obtained by measuring the distance between the two central points of the cross-section images at seven levels (P1-P7) selected in advance: 1-, 2-, 3-mm distance from the apical foramen, the curved vertex, the initial point of bending, root canal orifice and the midpoint between the canal orifice to the initial point of bending (Fig. 3).

#### Evaluation of cleaning ability by scanning electron microscopy (SEM)

After micro-CT scanning, two shallow grooves were created longitudinally on the buccal and lingual sides of the root. The grooves should run parallel to the curve of the root canal and not puncture it. Then the root was split in half using a hammer and chisel. The root canal orifices were blocked by sterile small cotton balls to prevent the contamination of canals throughout the process. Freeze-drying at − 80 ℃ for 24 h (Lyo Quest, Telstar, Spain), the split roots were sprayed with gold for the following SEM examination.

**Fig. 4:**
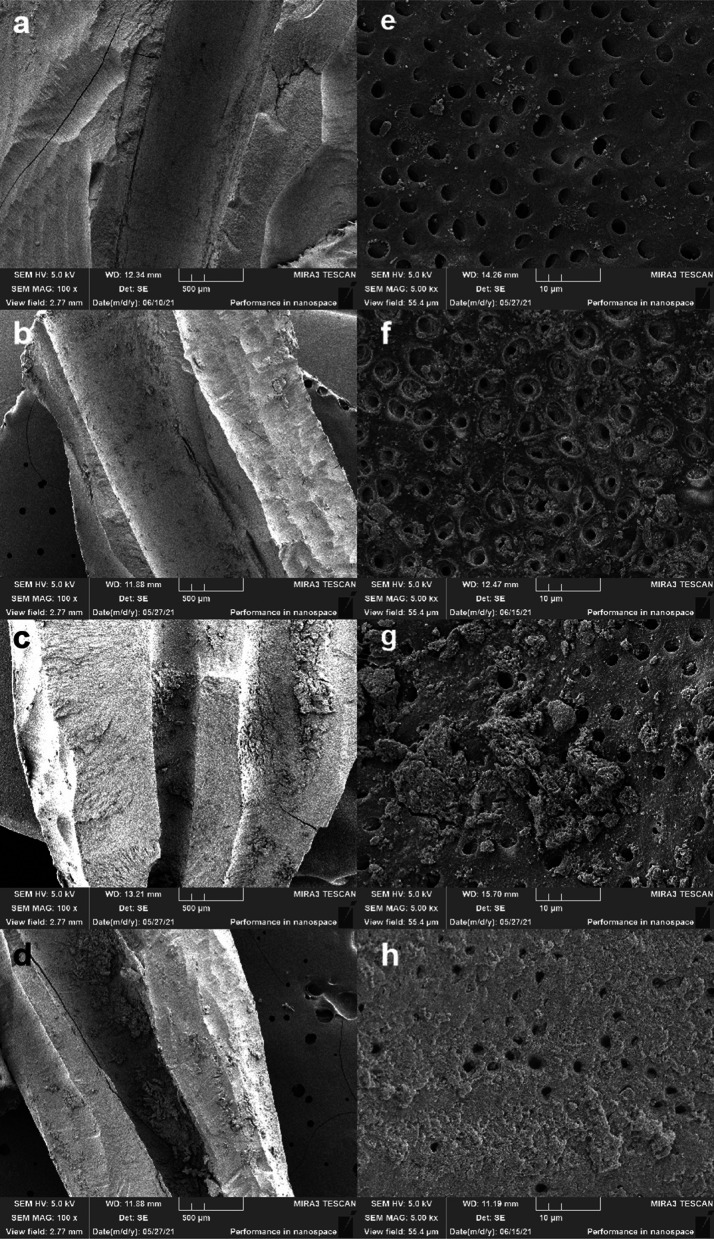
Representative SEM photos of debris (× 100) and smear layer (× 5000). **a** debris score 1; **b** debris score 2; **c** debris score 3; **d** debris score 4; **e** smear layer score 1; **f** smear layer score 2; **g** smear layer score 3; **h** smear layer score 4

Both the two parts of all canals were assessed, and two fields of the canal walls were randomly selected, respectively, in the coronal, middle, and apical thirds to be photomicrographed under × 100 and × 5000 magnification. The debris and smear layer in each region were scored using the scoring system proposed by Gutmann [19]. The debris was given a score of 1–4 according to the proportion of the surface covered by debris. The smear layer was scored based on the area covered by the smear layer and whether the dentinal tubules were visible and patent. The measurements were carried out independently by two researchers blinded to group allocation, one of whom had not participated in the study design or operation. Training and agreement on criteria has been achieved prior to scoring, and once the researchers have different opinions, an accordant score will be reached after discussion. The scoring example photos were shown in Fig. 4.

### Statistical analysis

The normalcy and homogeneity of variance of the data were checked by the Shapiro-Wilk test and Levene test. One-way analysis of variance (ANOVA) and the Tukey test were used to compare the 5 groups for variables that presented normal distribution (the angle of curvature, apical size, %UTA, %VI, AD). When the variables did not have a normal distribution (CT and SEM scores), the Kruskal–Wallis test was used to analyze the data, and the Mann–Whitney U test was used for multiple comparisons. The level of significance was set at 5%. Statistical Package for the Social Sciences v.21.0 (SPSS, IBM Brasil, SP, Brazil) and GraphPad Prism v.7.04 (GraphPad Software, America) was used for all analysis.

## Results

### Apically extruded debris, volume increment of canals, and untouched surface areas

No statistical differences were observed among the five groups regarding the apically extruded debris, the percentage of increment in root canal volume, and the percentage of untouched surface areas (*P* > 0.05). The details of the data were presented in Table 3.

**Table 3:** The details of % volume increment, % untouched surface area, and apically extruded debris (n = 12)

System	UTA (%)	AD (mg)	Volume (mm^3^)		
			Before	After	ΔV (%)
M3-L	3.66 ± 3.63	57.87 ± 22.36	8.54 ± 5.65	11.26 ± 4.37	82.40 ± 33.43
Reciproc Blue	4.90 ± 4.94	42.38 ± 21.58	9.51 ± 4.81	12.33 ± 4.85	36.96 ± 9.20
V-Taper 2H	5.65 ± 2.70	58.28 ± 21.78	8.71 ± 4.21	10.57 ± 3.96	28.72 ± 7.60
WaveOne Gold	3.64 ± 2.33	24.95 ± 7.2	8.09 ± 3.04	11.92 ± 2.08	60.22 ± 16.11
XP-endo Shaper	4.50 ± 2.91	30.78 ± 9.15	8.26 ± 5.72	11.49 ± 3.12	36.83 ± 9.23
P value (ANOVA)	0.56	0.57	0.97	0.97	0.21

Parameters normally distributed are presented with Mean ± SD.

### Canal transportation

Table 4 displayed the results of canal transportation. The differences in the amount of canal transportation were not significant between the five groups at the straight section (P6–P7) of canals and 1, 2, 3-mm levels from the apex (P1–P3), whereas XP showed less canal transportation than ML (*P* < 0.05) at the level of the curve vertex (P4), and at the level of the initial point of bending (P5), the centering ability of VT was better in comparison to WOG (*P* < 0.05).

**Table 4:** The deviation of root canal transportation (mm) (n = 12)

Measuring levels	M3-L	Reciproc Blue	V-Taper 2H	WaveOne gold	XP-endo shaper	*P* value
P1	0.10 ± 0.02 (0.08, 0.05–0.14)	0.08 ± 0.02 (0.06, 0.03–0.12)	0.11 ± 0.02 (0.09, 0.04–0.18)	0.12 ± 0.02 (0.11, 0.05–0.19)	0.08 ± 0.02 (0.05, 0.02–0.11)	0.77
P2	0.12 ± 0.02 (0.12, 0.07–0.16)	0.16 ± 0.04 (0.10, 0.04–0.12)	0.16 ± 0.04 (0.06, 0.02–0.15)	0.010 ± 0.04 (0.08, 0.06–0.14)	0.08 ± 0.02 (0.07, 0.04–0.14)	0.48
P3	0.11 ± 0.02 (0.07, 0.04–0.18)	0.07 ± 0.02 (0.04, 0.02–0.12)	0.07 ± 0.02 (0.05, 0.02–0.13)	0.11 ± 0.02 (0.08, 0.06–0.17)	0.07 ± 0.02 (0.04, 0.03–0.12)	0.56
P4	0.19 ± 0.05^a^ (0.10, 0.08–0.30)	0.08 ± 0.03 (0.07, 0.02–0.14)	0.10 ± 0.04 (0.05, 0.04–0.15)	0.09 ± 0.02 (0.08, 0.06–0.14)	0.07 ± 0.02^b^ (0.05, 0.03–0.10)	0.03
P5	0.16 ± 0.05 (0.06, 0.05–0.28)	0.12 ± 0.02 (0.07, 0.06–0.16)	0.08 ± 0.03^a^ (0.05, 0.02–0.13)	0.20 ± 0.04^b^ (0.22, 0.11–0.29)	0.10 ± 0.02 (0.08, 0.05–0.17)	0.04
P6	0.16 ± 0.05 (0.11, 0.06–0.26)	0.10 ± 0.02 (0.08, 0.05–0.15)	0.10 ± 0.01 (0.11, 0.07–0.12)	0.16 ± 0.03 (0.14, 0.09–0.23)	0.09 ± 0.03 (0.07, 0.03–0.15)	0.33
P7	0.18 ± 0.05 (0.13, 0.07–0.29)	0.15 ± 0.03 (0.13, 0.08–0.22)	0.14 ± 0.05 (0.08, 0.02–0.26)	0.20 ± 0.05 (0.18, 0.11–0.31)	0.13 ± 0.05 (0.05, 0.02–0.24)	0.17
*P* value	0.70	0.13	0.69	0.28	0.96	

P1–P3: 1-, 2-, 3-mm from apical foramen; P4: curved vertex; P5: initial point of bending; P6: The midpoint of P5 and P7; P7: root canal orifice.

Parameters non-normally distributed are presented with Mean ± SE (median, range)

No statistically significant difference was found within groups (Kruskal–Wallis test and Mann–Whitney test, *P* > 0.05 )

“a”and “b”: different letters in the same row indicated a statistically significant difference between groups (Kruskal–Wallis test and Mann–Whitney U test, *P* < 0.05 )

### SEM scores of debris and smear layers

**Fig. 5:**
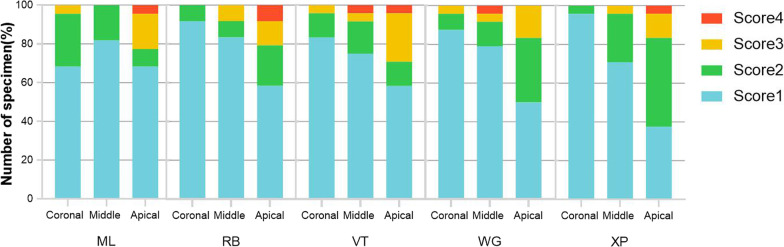
The proportion of specimens registered for each score in the evaluation of debris

**Fig. 6:**
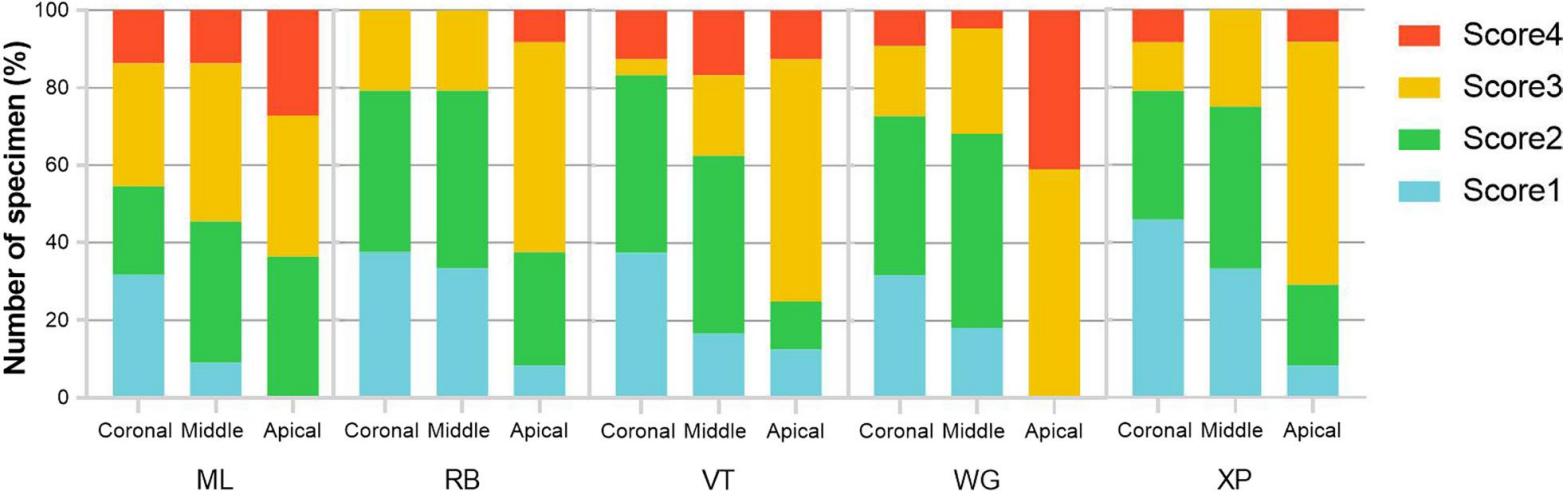
The proportion of specimens registered for each score in the evaluation of smear layers

The kappa values for the inter-researcher agreement were 0.87. The SEM scores of the debris and smear layer were shown in Figs. 5 and 6, respectively. All specimens had debris and smear layer on canal walls, and the apical thirds of the canals in all groups exhibited higher SEM scores than the coronal thirds (*P* < 0.05). In all three thirds, there were no significant differences in debris scores between groups, but in the apical thirds, RB and XP showed fewer smear layer scores than WOG (*P* < 0.01 and *P* < 0.05, respectively), and no statistical difference was found between the other groups in the middle and coronal thirds.

## Discussion

The shaping ability and cleaning effectiveness of single-file instruments have been demonstrated in previous studies [20], where the single-file systems performed similarly to rotary full-sequence NiTi systems using one instrument. The present study compared the apical debris extrusion, shaping, and cleaning ability of five advanced single-file systems with different metallurgy, instrument design, as well as kinematic strategy during severely curved canals preparation.

The extrusion of apical debris was associated with post-operative pain or discomfort, so the reduction in debris extrusion is one of the important factors to evaluate the outcomes of root canal preparation. NaOCl was used as the flushing fluid in this study despite researchers suggesting that NaOCl solution leaking from the apical foramen will interfere with the measurement of apically extruded debris [21]. This is because the substance extruded from the root apex contained intracanal irrigants in addition to necrotic tissue and dental debris during in vivo endodontic operations [22]. Furthermore, a 2% NaOCl solution can better simulate the procedure in the clinic when investigating the cleaning ability of the files. It is worth noting that ethylene diamine tetracetic acid (EDTA) was not employed in this study since it can chemically react with NaOCl to generate precipitates [23], which will compromise the accuracy of the measurement of AD.

All instruments generated debris extrusion more or less through the apical foramen with no discernible difference between the five systems. This was consistent with a previous study in which XP and RB extruded similar hard tissue debris in extracted maxillary molars [24]. However, De Deus et al. found that reciprocating files caused greater debris extrusion apically in comparison to rotary instruments [25]. It has been speculated that the instruments with reciprocating motion are better at squeezing debris into the spiral flutes and carrying the debris out of the root canal orifice, thereby reducing apical extrusion of debris. Rotary instruments, on the other hand, are more likely to produce a spiral effect which may force the debris out of the apical foramen. This tendency was not observed in the present study, which may be attributed to other characteristics of the instruments such as the cross-section and taper design. The unique S-shaped design of XP makes it act like a spoon during movement, providing unparalleled debris removal; The ML instrument has a unique longitudinal cutting plane on the traditional cross-section, which increases the debris discharge space by 20%; and the VT instrument has the smallest taper design and produces less dentin debris, potentially resulting in less apical extrusion. As a result of the combined influence of multiple aspects, there were no significant differences in AD among the five systems.

The micro-CT has been used in the evaluation of the shaping ability of endodontic instruments in recent years due to its precision and nondestructive ability, and the smaller the pixel size, the greater the accuracy of the evaluation [26]. Hence, the present study chose a 9 μm pixel size at which the anatomical details of the root canal could be observed. For three-dimensional volumetric assesment of the pulp space, several 3D analysis softwares have been developed to reconstruct two-dimensional data into three dimensions and analyze it qualitatively and quantitatively, such as OsiriX MD, 3D Slicer, and Mimics. The accuracy of them has been verified in many papers compared with real measurements [27–29], and the Mimics software was used in the present study. According to the findings, the volumes of canals in the five systems were similar at baseline, with no significant difference among groups. This was crucial for ensuring the validity of the results and reducing bias by confirming the work of the five systems under identical conditions. The percentage of volume increment was shown to be equal among the five systems, which was different from the study of Caviedes et al., who found significant differences of canal volume increment: RB > WOG > XP [30]. This may be related to the fact that the specimens they chose were lower premolars with a straight canal, indicating that the performance of instruments in volume increment may be affected by the curvature of the canals.

On average, the percentages of untouched root canal areas of the five systems (ML, RB, VT, WOG, and XP group) were 3.66%, 4.90%, 5.65%, 3.64%, and 4.50%, respectively, without any statistical difference among groups. These values were less than those reported in other studies, which showed that the untouched canal areas varied from 9.6 to 47.6% [31–33]. The difference might be attributed to the fact that the root canal samples in this study were nearly round-shaped, with an average initial file size of 9–10. A large number of untouched areas is more likely to be observed in the root canal walls of oval or broad canals. Therefore, more studies should be conducted to evaluate these parameters in diverse canal morphologies. Despite the advances in instruments and the regular shape of canals, none of the systems could prepare all the canal walls in severely curved canals, underlining the essential role of chemomechanical preparation via irrigation strategies.

The measurement method of canal transportation was modified according to previous studies [34–37]. Since the uncertainty of the direction in canal transportation, the direct measurement of central points is considered more telling. With this measuring method, it was found that the centering ability of five instruments were similar at the 1-, 2-, and 3-mm levels from the apical foramen and the straight section of canal. The canal transportation occured mainly at the level of the curved vertex and the initial point of bending with significant differences: XP showed less canal transportation than ML at the level of the curved vertex (*P* < 0.05), and VT resulted in better centering than WOG at the initial point of bending (*P* < 0.05). This agreed with another study that reported XP generated less canal transportation than WOG at the 7-mm level using the micro-CT method [38]. Moreover, Shenoi et al. also demonstrated the superior centering ability of VT compared to ProTaper Next, and HyFlex CM in curved canals [39]. Gundappa et al. found that the taper was one of the main factors related to the occurrence of canal transportation in a meta-analysis of the centering ability of NiTi instruments [40]. Instruments with small taper had good flexibility, which could better maintain the original axial direction of the root canal, reducing canal transportation but resulting in less dentin removal. The XP instrument is manufactured from Max-wire with a fixed small taper of 0.01, which endows it with super-elasticity and extreme flexibility. It has a “Booster tip” design, and this non-cutting tip with six cutting edges can provide optimal guidance for the instrument and maintain the original curvature when passing through root canals. The VT instrument is a CM wire-based NiTi instrument with superior flexibility and resistance to cyclic fatigue. It has a variable taper design like WG, RB, and ML, but its apical taper is smaller than the other three systems. It’s possible that the larger apical taper and more stiff tips of WG and ML contribute to their increased canal transportation. Nevertheless, according to Wu et al., apical canal transportation of less than 0.3 mm would have the least impact on the prognosis of root canal therapy [41]. Under this criterion, the canal transportation of the instruments assessed in this study was acceptable and the differences between systems were negligible.

The smear layer is a kind of “muddy” material that clings to the canal walls as a result of the rasping and trowelling actions of instruments in root canal preparation, including fragments of dentin, microorganisms, and necrotic tissue. It has been reported that the residual debris and smear layer on the canal walls can affect the success rate of root canal therapy. The possible mechanism is to obstruct the penetration of disinfectants and the contact between root canal walls and filling paste [42–44]. In the present study, all specimens had debris and smear layer on canal walls in different groups, and the results agreed with previous findings that the apical thirds of the canal presented more amount of debris and smear layer than the coronal thirds in all systems [45, 46]. Overall, the root canal walls were fairly clean, particularly in terms of debris, which might be attributed to the physical irrigation. The smear scores were generally higher, which may be connected to the lack of chemical irrigation in our study, because the smear layer is adhered to the canal wall, it’s hard to be washed off by simple physical irrigation without the supplementary use of chemical irrigation. In practical situations, a combination of EDTA and NaOCl could achieve a better chemical preparation effect. Nonetheless, the outcomes of the present study were comparable since this experiment was a contrast experiment of the five instruments and the irrigation strategies were the same.

There were no significant differences in debris scores across groups on the entire surface of canals. As for the smear scores, no significant differences were found in the middle and coronal thirds, but in the apical thirds, RB and XP were shown to have fewer smear scores than WOG (*P* < 0.01 and *P* < 0.05, respectively). It has been concluded that the cutting efficiency [47] and cross-sectional area [48] of the files play significant roles in the production of debris and smear layer. This may explain the difference between RB and WOG, which are both subjected to special heat treatment with different cross-section designs. The WOG file has an offset parallelogram geometry leaving one cutting edge in contact with the canal wall, which reduces the risk of taper lock but may lead to less scraping of canal walls. The cross-section of RB is S-shaped with two cutting edges, which is highly efficient in cutting and has a smaller cross-section area than WOG. Therefore, sharp cutting edges and a larger chip space may aid in the elimination of the smear layer. The superior cleaning ability of XP may be related to its unique design. Because of the peculiarity of Max-wire, XP can change its morphology into a semi-circular spoon shape when rotating. Then it can protrude to the canal wall and expand or contract according to the shape of the root canal to obtain an appropriate cleaning of the canal walls. Besides, the ML file has a double S-shaped cross-section, and the VT file has a modified convex triangle cross-section with three cutting edges, both are smaller than that of WOG, therefore their SEM scores were lower on average than WOG, but the difference was not significant. To our knowledge, there have been no SEM analyses of the cleaning efficiency of ML and VT until now, so no comparison could be made.

## Conclusion

Within the limitations of this ex vivo study, it can be concluded that the newly introduced NiTi instruments XP-endo Shaper, V-Taper 2H, and M3-L performed equally to WaveOne Gold and Reciproc Blue in apically debris extrusion, dentin removal, and untouched root canal areas during the preparation of severely curved canals. None of the instruments were able to implement perfect centering and cleaning of root canals, however, XP-endo Shaper and V-Taper 2H resulted in less canal transportation than M3-L and WaveOne Gold at the curved section. Reciproc Blue and XP-endo Shaper were associated with less smear layer compared to WaveOne Gold in the apical thirds. Further research will be required to verifiy these results in the future.

## Supplementary Information

**Additional file 1.** **The device used to weigh the debris and its calibration**.

## Data Availability

The datasets used and/or analyzed during the current study are available from the corresponding author on reasonable request.

## Abbreviations

VI: Volume increment of root canals
UTA: Untouched root canal areas
CT: Canal transportation
AD: Apically extruded debris
SEM: Scanning electron microscope
WOG: WaveOne Gold
RB: Reciproc Blue
XP: XP-endo Shaper
VT: V-Taper 2H
ML: M3-L
WL: Working length
CEJ: Cementoenamel junction
EDTA: Ethylene diamine tetracetic acid.

## Declarations

The human molar teeth used in the present study were all isolated teeth extracted at the Stomatological Hospital of Wuhan University for reasons unrelated to the study. Because it is impracticable to associate each tooth with the patient and contact them to gain informed consent, the need of informed consent requirement was waived with the approval of the Ethics Committee of the School and Hospital of Stomatology, Wuhan University, China.
