# Supplementary material for: Comparison of five single-file systems in the preparation of severely curved root canals: an ex vivo study
Source: BMC Oral Health. 2022 Dec 28;22:649. doi: 10.1186/s12903-022-02668-3 (PMC9798582; doi:10.1186/s12903-022-02668-3)
Supplement: Supplementary file 1 — Additional file 1. The device used to weigh the debris and its calibration. [file 12903_2022_2668_MOESM1_ESM.docx]

**The** **device used to weigh the debris and its calibration:**


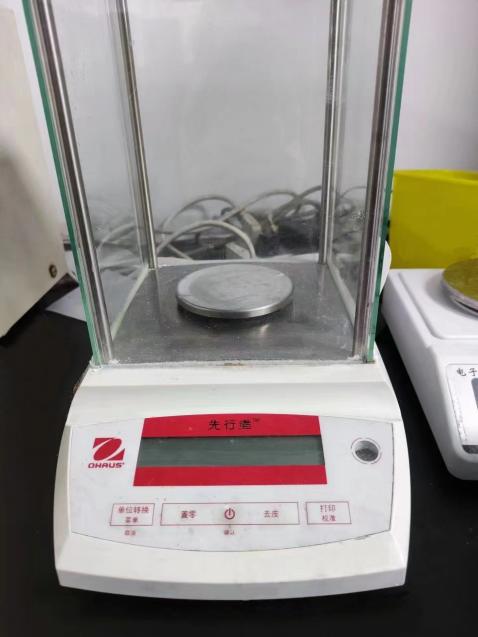

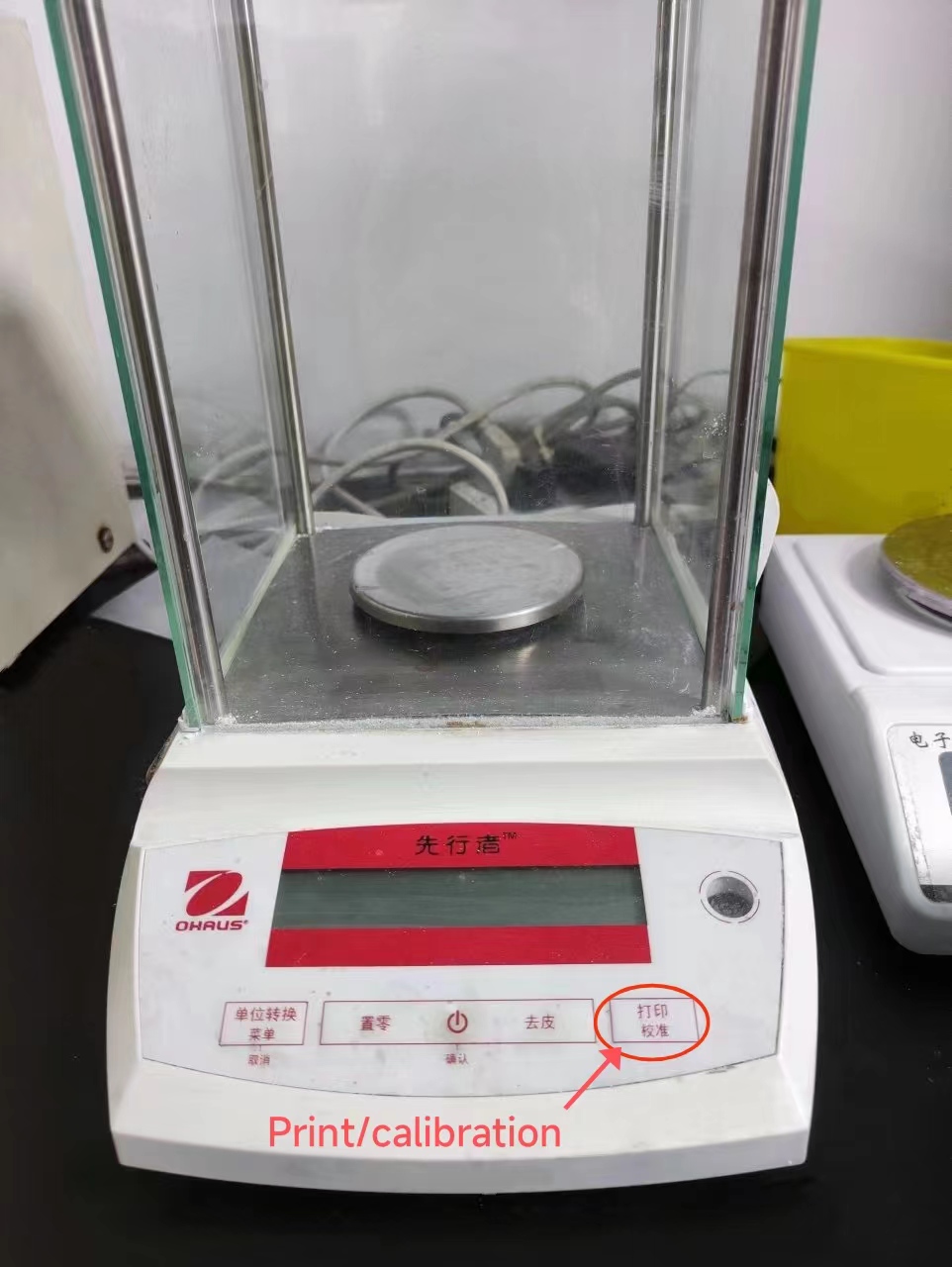


Fig 1. The [precision](javascript:;) [balance](javascript:;) used to weigh Fig 2. The calibration button for

the debris (CP214C,OHAUS , America). automatic internal calibration.


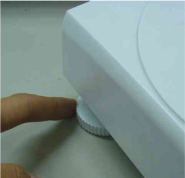

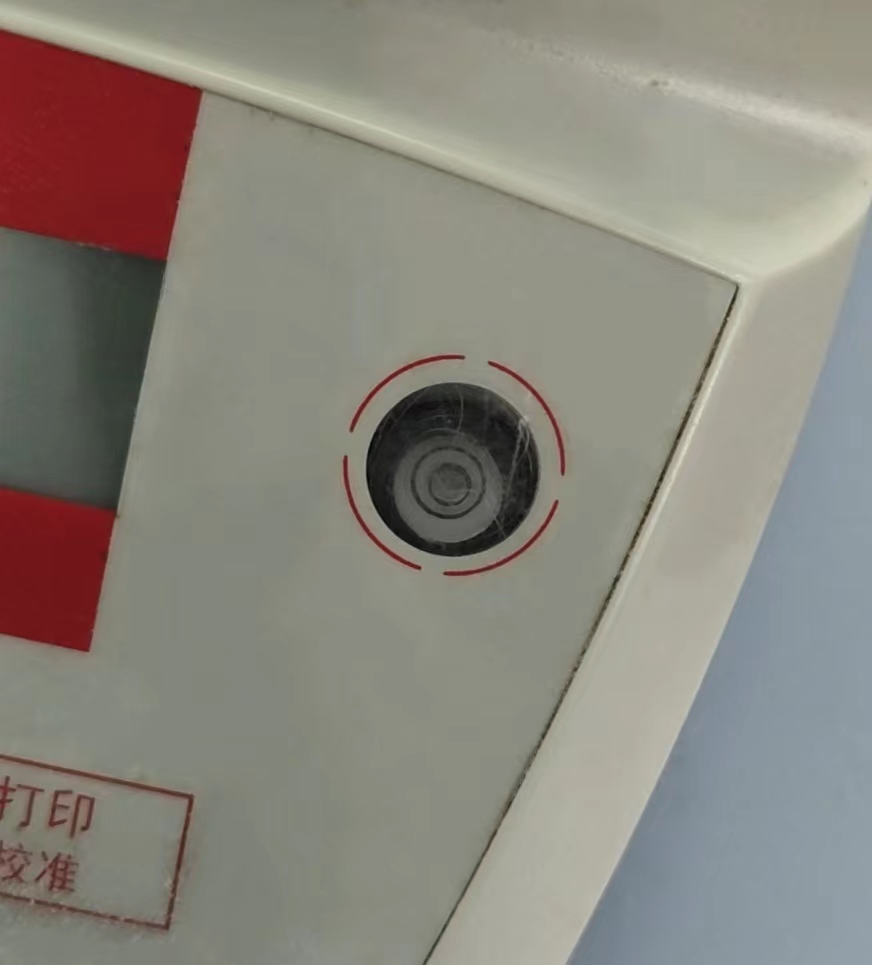


Fig 3. Adjust the front-mounted horizontal adjustment bubble to achieve horizontal balance.
